# Non-linear association between serum vitamin D and bacterial vaginosis in U.S. women: analysis from NHANES 2001–2004

**DOI:** 10.3389/fnut.2025.1606246

**Published:** 2025-05-21

**Authors:** Ming Liu, Jingyu Xiang, Miao Zhang, Yifang Zhang, Jian Liu

**Affiliations:** Department of Gynecologic Oncology, The First Affiliated Hospital of Bengbu Medical University, Bengbu, China

**Keywords:** serum vitamin D, bacterial vaginosis, nutrients, NHANES, cross-sectional study

## Abstract

**Background:**

Previous studies have reported inconsistent associations between serum vitamin D levels and bacterial vaginosis (BV). Therefore, this study aimed to comprehensively investigate the relationship between serum vitamin D concentrations and BV risk among adult women in the United States.

**Methods:**

Data from the National Health and Nutrition Examination Survey (NHANES) 2001–2004 were analyzed. Multivariate logistic regression was used to assess the association between serum vitamin D levels and BV. Additionally, smoothing curve fitting and subgroup analyses were performed to explore potential non-linear relationships and effect modifications.

**Results:**

A total of 1,397 adult women were included. After adjusting for multiple covariates, serum vitamin D levels showed a significant non-linear negative association with BV risk. Subgroup analyses revealed that this association was not statistically significant among certain ethnic groups and women with lower body mass index (BMI).

**Conclusion:**

Serum vitamin D levels were non-linearly and negatively associated with the risk of bacterial vaginosis in adult women in the United States. Notably, the association was no longer present at serum vitamin D concentrations ≥63.1 nmol/L, which may inform future research.

## 1 Introduction

Bacterial vaginosis (BV) is a common vaginal infection caused by an imbalance in the vaginal microbiota and is characterized by a decrease in the number of lactobacilli together with an overproliferation of anaerobic bacteria (e.g., *Gardnerella vaginalis*, Prevotella, and *Atopobium vaginae*) (1). This microecological imbalance causes the vaginal pH to rise, disrupting the normal acidic environment and leading to a range of symptoms (2). Typical symptoms in about 50% of patients are a thin, grayish-white, or yellowish discharge with a fishy odor. This is especially common after sexual intercourse (3, 4). BV can cause many complications, including an increased risk of sexually transmitted infections (5–8), such as human immunodeficiency virus (HIV), herpes simplex virus (HSV), *Neisseria gonorrhoeae*, and *Chlamydia trachomatis*, and cause genitourinary infections in women and adverse pregnancy outcomes (9–12). In addition, BV can have an impact on the mental health and social quality of women’s lives (13, 14).

The etiology of BV is not fully understood, and the recurrence rate is high. One study showed a recurrence rate of 58 percent within 12 months of standard treatment with metronidazole (15). This high recurrence rate may be due to a number of factors, including bacterial biofilm formation, antibiotic resistance, incomplete restoration of the vaginal micro-ecosystem, and unsafe sexual practices (16–19). Therefore, existing treatments have limited effectiveness in preventing recurrence of BV, and there is an urgent need to explore new treatment strategies.

Vitamin D is a fat-soluble nutrient that is synthesized by the skin, mainly through exposure to sunlight, but also through diet and supplementation, and has the function of promoting calcium and phosphorus absorption, supporting cardiovascular health, regulating cell growth and differentiation, and immunity (20, 21). The role of vitamin D in female reproductive health has recently attracted increasing attention. Studies have shown that serum vitamin D may influence the development and progression of BV in several ways, such as exerting direct antibacterial effects, preventing pathogen biofilm formation, regulating immune and inflammatory responses, maintaining the acidic vaginal environment, and reprogramming epigenetics and metabolism (22–25). Although several studies have focused on the relationship between serum vitamin D and the risk of BV, the results in the existing literature are inconsistent. Turner et al. conducted a randomized controlled trial that showed that high-dose vitamin D supplementation in addition to standard metronidazole therapy did not reduce BV recurrence (26). Subsequently, in their study of Zimbabwean women of childbearing age, they showed that serum vitamin D insufficiency was not associated with increased prevalence or incidence of BV (27). However, some studies support the idea that people with vitamin D deficiency or insufficiency are more likely to develop BV (28, 29), and that vitamin D supplementation may be beneficial in treating BV (30). Therefore, further studies are needed to test the association between serum vitamin D and BV.

## 2 Materials and methods

### 2.1 Survey description

All data for this study came from the National Health and Nutrition Examination Survey (NHANES), conducted by the Centers for Disease Control and Prevention (CDC). The survey is highly credible and informative in academic research because it uses probability sampling to collect data. The data source for this analysis was the 2001–2002 and 2003–2004 cycles of NHANES. All participants provided written informed consent, which was approved by the Ethics Review Board of the National Center for Health Statistics (NCHS). Comprehensive NHANES data and research methods are publicly available at https://www.cdc.gov/nchs/nhanes/.

### 2.2 Study population

There were 21,161 participants in NHANES 2001–2004. After excluding males (n = 10,301) and those with missing data for bacterial vaginosis (n = 8,816), serum vitamin D (n = 104), smoking (n = 324), drinking (n = 134), BMI (n = 15), and PIR (n = 70), 1,397 participants were included in the study (Supplementary Figure 1).

### 2.3 Study variables

#### 2.3.1 Diagnostic for bacterial vaginosis

The vaginal swab specimens were processed, stored, and transported to the Magee-Women’s Hospital in Pittsburgh, Pennsylvania. After Gram staining in the laboratory, slides were scanned with a low-power objective to locate clusters of epithelial cells, followed by quantification of the mean number of Lactobacillus forms, Gardnerella spp., anaerobic Gram-negative bacilli, and Mobiluncus forms. Using the Nugent method, we calculated the BV score and considered it BV positive when the total number of bacteria reached 7–10.

#### 2.3.2 Serum vitamin D

Serum vitamin D levels were measured using the DiaSorin RIA kit (Stillwater, MN). According to the Endocrine Society’s 2024 Clinical Practice Guidelines (31), the traditional thresholds used to define vitamin D deficiency, insufficiency, and sufficiency are no longer widely used due to the lack of sufficient evidence demonstrating a clear association between different serum 25(OH)D levels and clinical outcomes. Historically, these thresholds were primarily based on bone health and were defined as deficiency (<50.00 nmol/L), insufficiency (50.00–74.99 nmol/L), and sufficiency (≥75.00 nmol/L) (32). In this study, we retain this classification for consistency with previous literature but note that these thresholds may not fully reflect the role of vitamin D in mucosal immunity, which may require different serum concentration levels.

#### 2.3.3 Covariates

Covariates included age, race, education, marital status, PIR, BMI, smoking (defined as having smoked at least 100 cigarettes in life), and alcohol consumption (defined as 12 or more drinks per year). Participants’ age was categorized as <35 and ≥35 years. Marital status was categorized as married/living with a partner, never married, divorced/separated/widowed. PIR was categorized into three groups: <1.3, 1.3–3.49, and ≥3.5. BMI was categorized into four groups: <18.5 kg/m^2^, 18.5–24.99 kg/m^2^, 25–29.99 kg/m^2^, and ≥30 kg/m^2^. All detailed data collection processes and measurement procedures for these covariates are available on the National Center for Health Statistics (NCHC) website.

### 2.4 Statistical analysis

In this study, the complex multi-stage sampling weighting guidelines provided by NHANES were followed. The baseline table describing the study population was divided according to BV positivity and negativity, with continuous variables expressed as weighted mean and standard deviation (SD) and categorical variables expressed as percentages. We used the t-test and chi-squared test to compare the distribution of baseline characteristics between groups. To further explore the relationship between serum vitamin D and BV, multivariate logistic regression models were constructed: the crude model was not adjusted for any covariates; model 1 was adjusted for age and race; and model 2 added education, marital status, PIR, BMI, smoking, and drinking to model 1. In addition, a smooth curve fit graph was plotted for model 2 to verify the non-linear relationship between serum vitamin D and BV, and a segmented linear regression model was used to further investigate potential threshold effects. Finally, we performed subgroup analyses and interaction tests stratified by covariates to control for confounding bias and enhance the reliability of the results. All data were processed and analyzed using R (version 4.4.2) and Empower Stats (version 2.0). *p* < 0.05 was considered statistically significant.

## 3 Results

### 3.1 Baseline characteristics

Of the 1,397 female subjects aged 20 years and older with complete data included in the study, 590 were BV positive (42.23%) and 807 were BV negative (57.77%). Supplementary Table 1 summarizes the baseline demographic characteristics weighted by BV status. Significantly higher proportions of BV-positive individuals were Black, had a high school education or less, lived alone, and were smokers compared with BV-negative individuals. In addition, BV-positive women had a higher BMI, while their PIR and serum vitamin D levels were lower. No significant differences were found between the two groups with regard to age and alcohol consumption (*p* > 0.05). Supplementary Table 1 also shows the percentage of those with adequate, inadequate, and deficient serum vitamin D levels in the two groups, which showed that the percentage of those with adequate serum vitamin D level was significantly lower in the BV-positive group (*p* < 0.0001).

### 3.2 Associations between vitamin D and risk of bacterial vaginosis

The association between serum vitamin D levels and BV is shown in Supplementary Table 2. The results of multiple logistic regression analysis showed that the risk of BV decreased with increasing serum vitamin D levels. After adjustment for age and race, this association remained significant (OR = 0.99, 95% CI: 0.98–0.99, *p* < 0.0001), i.e., for every 1 nmol/L increase in serum vitamin D, there was a 1% reduction in the risk of developing BV. In model 2, although the association was not significant, there was still a trend towards a negative association between serum vitamin D levels and the risk of BV. In addition, participants were divided into deficiency (<50.00 nmol/L), insufficiency (50.00–74.99 nmol/L), and sufficiency (≥75.00 nmol/L) groups based on serum vitamin D levels, and the sufficiency group was used as the reference for analyses. The results showed that individuals with serum vitamin D insufficiency had a significantly increased risk of developing BV compared with the sufficiency group, whereas the insufficient individuals did not have a significantly increased risk.

Smoothed curve fitting based on model 2 showed a non-linear negative correlation and a possible saturation effect between serum vitamin D and BV (Supplementary Figure 2). Threshold effect analysis using a two-segment piecewise linear regression model indicated that the minimum threshold for a protective effect was 63.1 nmol/L (log-likelihood ratio < 0.001). On the left side of the inflection point, the prevalence of BV decreased progressively with increasing serum vitamin D. However, on the right side of the inflection point, the effect of serum vitamin D on the risk of BV was attenuated and may not even be clinically significant (Supplementary Table 3).

To assess the robustness of the association between serum vitamin D and BV risk, we performed subgroup analyses and tested for interactions between different populations, the results of which are shown in Supplementary Figure 3. Subgroups were stratified by age, race, education level, marital status, PIR, BMI, smoking, and alcohol consumption. Analysis showed that in most subgroups, serum vitamin D levels were negatively associated with the risk of BV (HR < 1) and were statistically significant (e.g., age < 35: *p* < 0.001; White: *p* = 0.0024; full details in Supplementary Figure 3). It is worth noting that the association between serum vitamin D levels and the risk of BV was not statistically significant in the subgroups of “other races” and “BMI < 18.5”. The results of the interaction test showed that there was no statistically significant difference in the interaction between subgroups (P-interaction > 0.05), suggesting that the negative association between serum vitamin D and the risk of BV is consistent in populations with different characteristics.

## 4 Discussion

Our study showed a non-linear negative association between serum vitamin D levels and the risk of BV, but this association was no longer present at serum vitamin D concentrations ≥ 63.1 nmol/L. This study systematically analyzed the causes of conflicting conclusions in previous observational studies, laying an evidence-based medical foundation for precise vitamin D supplementation strategies for people at high risk of BV.

We found that serum vitamin D deficiency was associated with an increased risk of BV, which is consistent with previous hypotheses about the mechanism by which vitamin D regulates mucosal immunity and maintains vaginal microecological balance. Holick et al. (33) pointed out that vitamin D promotes the expression of antimicrobial peptides by activating the vitamin D receptor (VDR), improves epithelial barrier function, and inhibits pathogen colonization. In addition, vitamin D may protect the vagina from bacterial infection by increasing the stability, proliferation and differentiation of stratified squamous epithelial cells through activation of the VDR, p-RhoA and p-Ezrin pathways, as suggested in other systems (34). Vitamin D can also induce the synthesis and secretion of insulin and increase the phosphorylation and inactivation of glycogen synthase kinase (inhibitor of glycogen synthesis) in adipose tissue, indirectly promoting glycogen production. This may alter vaginal glucose homeostasis, increasing the relative abundance of lactobacilli and lowering local pH (35–37).

Smooth curve fitting revealed a non-linear negative association between serum vitamin D and the risk of BV and identified a threshold effect of 63.1 nmol/L (log likelihood ratio <0.001). The inflection point at 63.1 nmol/L may reflect a biological saturation effect, with the inverse association between vitamin D and BV risk plateauing beyond this level. Its clinical relevance requires validation in prospective interventional trials. This threshold is significantly lower than the 75 nmol/L recommended by the Endocrine Society for bone health, suggesting that vitamin D’s role in mucosal immune protection may be saturated earlier than its effect on bone health. Specifically, when serum vitamin D levels are below 63.1 nmol/L, the risk of BV increases with decreasing vitamin D levels, while above this threshold the protective effect stabilizes. This difference may be due to the local metabolic characteristics of the vagina: 1α-hydroxylase expressed by vaginal epithelial cells can convert vitamin D in serum to the active form 1,25(OH)₂D, and it can effectively trigger the VDR signaling pathway at lower serum concentrations (32, 38, 39). However, the skeletal system has a higher requirement for vitamin D, and levels usually need to be maintained above 75 nmol/L to inhibit parathyroid hormone (PTH) secretion and maintain calcium and phosphorus homeostasis (32, 40). This finding provides an important basis for stratified intervention: for women at high risk for BV, the target for serum vitamin D optimization can be set at ≥63.1 nmol/L rather than following the standard for bone health uniformly. Maintaining serum vitamin D levels ≥63.1 nmol/L was associated with both a lower risk of bacterial vaginosis and a reduced likelihood of harm related to excessive supplementation. However, this threshold is an exploratory result that is not sufficient to establish clinical guidelines.

Subgroup analyses of this study showed that the negative association between serum vitamin D and the risk of BV remained robust (P-interaction > 0.05) in most stratifications, such as age, education level, and marital status, suggesting the broad biological effects. However, this link did not reach statistical significance in the subgroups certain ethnic groups and “BMI < 18.5.” This could be because of a small sample size or factors that were unique to those groups. It is worth noting that, as a fat-soluble vitamin, vitamin D is closely associated with adipose tissue in terms of metabolism, storage, and distribution (41). It’s harder to store vitamin D if there is a big loss of fat tissue, like when a person has a low BMI or cachexia (42). This could cause the results to be skewed. One possible explanation for the finding that the link was weaker in the “BMI < 18.5” subgroup is this mechanism.

The present study has the following limitations. First, while the biological plausibility of vitamin D’s immunomodulatory effects on vaginal microbiota remains temporally stable, our reliance on 2001–2004 NHANES data introduces potential contextual biases. Temporal shifts in environmental determinants—particularly evolving dietary patterns, sunscreen utilization trends, and lifestyle modifications—may alter population-level vitamin D status profiles. Furthermore, the cross-sectional design inherently precludes causal inference regarding vitamin D’s protective mechanisms against bacterial vaginosis (BV). Subsequent research should prioritize contemporary cohorts with standardized BV phenotyping to validate these findings in modern populations. Second, although the NHANES database reduces seasonal bias in vitamin D measurements by using a seasonally adjusted sampling scheme (e.g., balanced inclusion of samples tested in different seasons) (43), individual-level variations in sun exposure and dietary intake may still introduce residual confounding in the results. Third, there may have been a higher risk of type II error because the sample sizes for subgroups like “other races” (n = 57) and “BMI < 18.5” (n = 34) were not big enough. Fourth, even after accounting for important demographic and lifestyle factors, some unmeasured influences (like how often people have sex, use contraception, maintain vaginal hygiene, and their hormonal levels) might still affect the results (44). Future studies need to include prospective cohorts or randomised controlled trials (RCTs) to verify causality, while more confounders and dynamic monitoring of vitamin D levels are recommended.

## 5 Conclusion

In summary, this study supports an association between vitamin D insufficiency and an increased risk of BV, and this association is consistent in most subgroups of the population. The fact that this association is no longer significant when serum vitamin D concentrations reach or exceed 63.1 nmol/L provides an important basis for the development of precise intervention strategies.

## Data Availability

The datasets presented in this study can be found in online repositories. The names of the repository/repositories and accession number(s) can be found at: https://www.cdc.gov/nchs/nhanes/.
