# Supplementary material for: Non-linear association between serum vitamin D and bacterial vaginosis in U.S. women: analysis from NHANES 2001–2004
Source: Front Nutr. 2025 May 21;12:1606246. doi: 10.3389/fnut.2025.1606246 (PMC12133536; doi:10.3389/fnut.2025.1606246)
Supplement: Supplementary file 1 [file Table_1.docx]

Supplementary Material

# Supplementary Figures

**Figure 1.** Flowchart for participant inclusion.


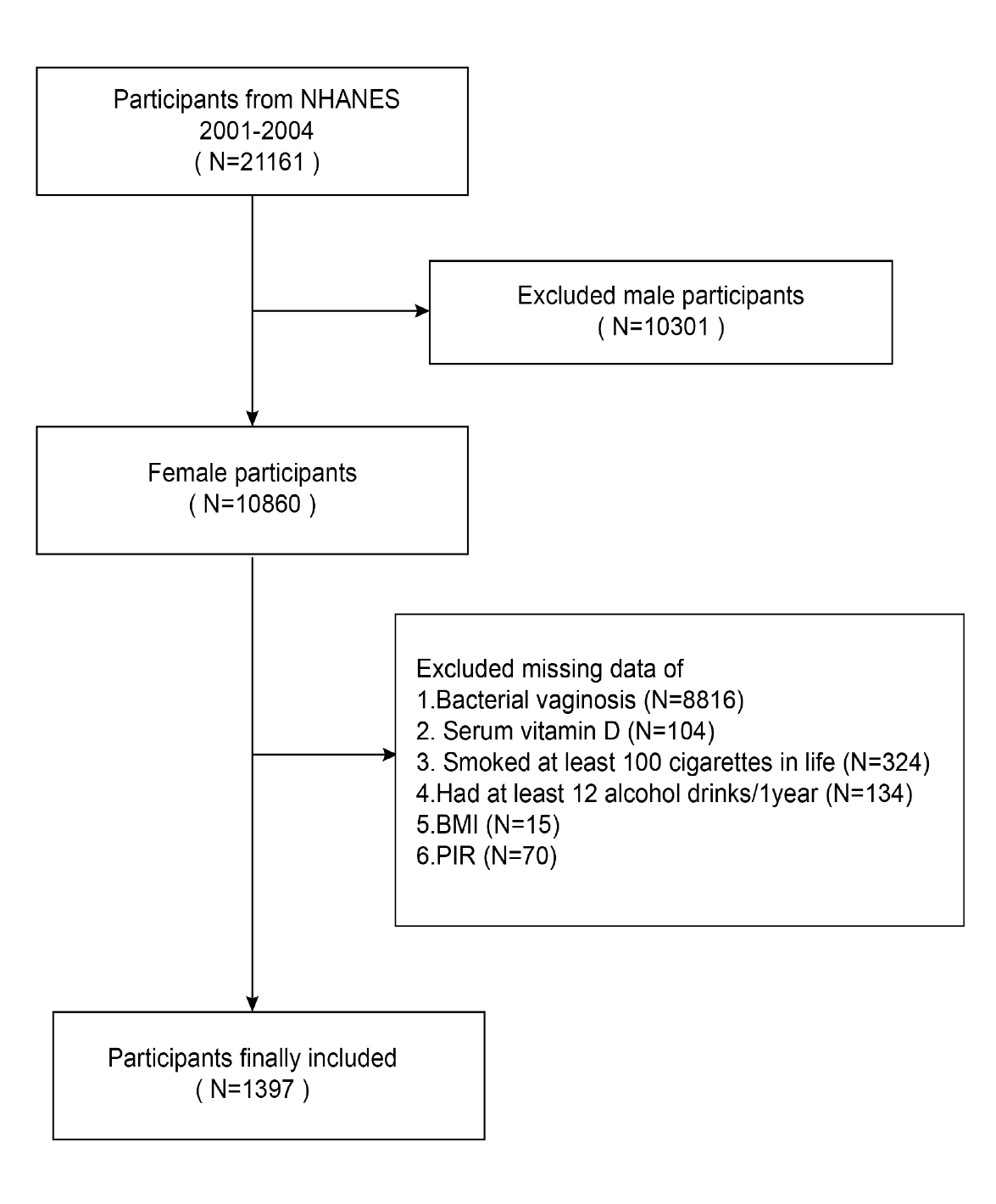


**Figure 2.** Assocication of serum vitamin D with probability of bacterial vaginosis after adjusted by model 2.


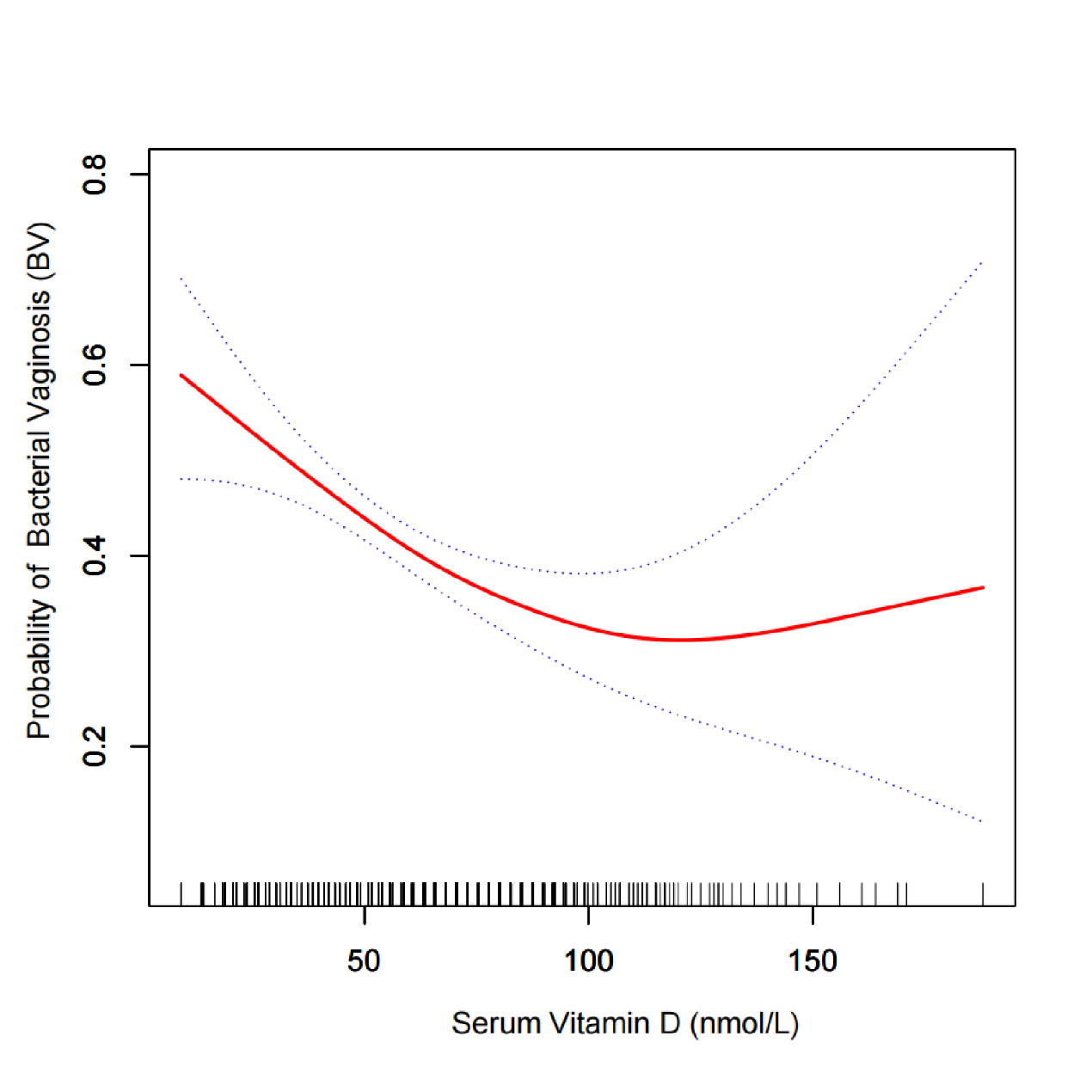


**Figure 3.** Subgroup analysis between serum vitamin D and bacterial vaginosis.


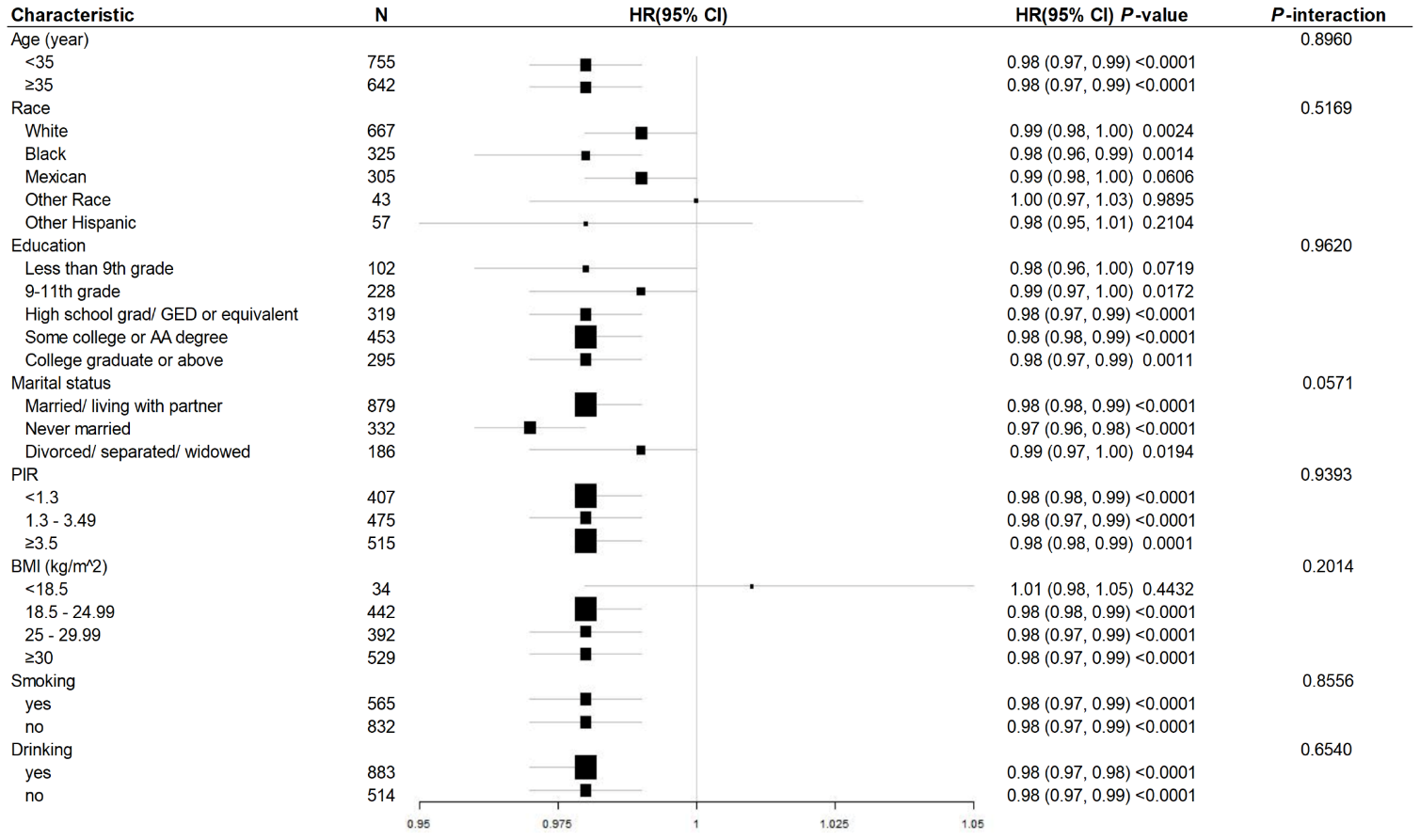


Note: The statistical power to detect significant differences is limited due to small sample sizes in some subgroups (e.g., BMI <18.5).

# Supplementary Tables

**Table 1.** Baseline characteristics of participants, stratified by the presence or absence of bacterial vaginosis (N = 1397).

| Characteristic | Bacterial vaginosis | | *P*-value |
| --- | --- | --- | --- |
|  | Negative | Positive |  |
| Number | 807 | 590 |  |
| Age (year) | 34.86±8.76 | 35.09±8.91 | 0.6416 |
| Race |  |  | <0.0001* |
| White | 75.31 | 56.34 |  |
| Black | 8.12 | 23.40 |  |
| Mexican | 8.18 | 9.19 |  |
| Other Race | 3.46 | 4.96 |  |
| Other Hispanic | 4.93 | 6.11 |  |
| Education |  |  | <0.0001* |
| Less than 9th grade | 2.97 | 3.97 |  |
| 9-11th grade | 10.25 | 15.74 |  |
| High school grad/ GED or equivalent | 21.13 | 27.40 |  |
| Some college or AA degree | 33.53 | 37.87 |  |
| College graduate or above | 32.12 | 15.02 |  |
| Marital status |  |  | <0.0001* |
| Married/ living with partner | 67.38 | 58.20 |  |
| Never married | 23.38 | 23.08 |  |
| Divorced/ separated/ widowed | 9.25 | 18.71 |  |
| PIR | 3.07±1.65 | 2.40±1.61 | <0.0001* |
| BMI (kg/m^2) | 27.39±7.10 | 29.09±7.66 | <0.0001* |
| Smoking |  |  | <0.0001* |
| yes | 38.38 | 52.07 |  |
| no | 61.62 | 47.93 |  |
| Drinking |  |  | 0.7225 |
| yes | 69.64 | 68.74 |  |
| no | 30.36 | 31.26 |  |
| Serum vitamin D (nmol/L) | 67.10±25.92 | 56.63±26.38 | <0.0001* |
| Serum vitamin D subgroups |  |  | <0.0001* |
| Deficient (<50 nmol/L) | 25.75 | 43.92 |  |
| Insufficient (≥50,<75 nmol/L) | 40.32 | 33.56 |  |
| Sufficient (≥75nmol/L) | 33.93 | 22.52 |  |

Continuous variables are presented as mean ± SD; P values were calculated using weighted linear regression models to compare group differences.
Categorical variables are presented as percentages; P values were calculated using weighted chi-square tests.
PIR: poverty income ratio; BMI: body mass index.
*P < 0.05 indicates statistical significance.

**Table 2.** Associations between the serum vitamin D and Bacterial vaginosis infection among women.

| Exposure | Crude Model | Model 1 | Model 2 |
| --- | --- | --- | --- |
|  | OR (95%Cl)  *P*-value | OR (95%Cl)  *P*-value | OR (95%Cl)  *P*-value |
| Serum vitamin D(nmol/L) | 0.98 (0.98, 0.98)  <0.0001 | 0.99 (0.98, 0.99)  <0.0001 | 0.99 (0.98, 1.00)  0.0004 |
| Serum vitamin D subgroups |  |  |  |
| Sufficient (≥75nmol/L) | 1.0 | 1.0 | 1.0 |
| Insufficient (≥50, <75 nmol/L) | 1.21 (0.90, 1.62)  0.2065 | 1.09 (0.81, 1.48)  0.5588 | 1.11 (0.81, 1.51)  0.5275 |
| Deficient (<50 nmol/L) | 2.98 (2.25, 3.94)  <0.0001 | 1.95 (1.42, 2.68)  <0.0001 | 1.77 (1.26, 2.48)  0.0009 |

Crude Model adjust for: none

Model 1 adjust for: age, race

Model 2 adjust for: age, race, education, marital status, PIR, BMI, smoking, drinking

**Table 3.** A threshold effect analysis of the relationship between serum vitamin D and bacterial vaginosis was performed using a two-segment piecewise linear regression model.

|  | Bacterial vaginosis  *β* (95% CI) *P*-value |
| --- | --- |
| Fitting by standard linear model | 0.98 (0.98, 0.98) <0.0001 |
| Fitting by two-segment piecewise linear model |  |
| Inflection point | 63.1 |
| < Inflection point | 0.97 (0.96, 0.98) <0.0001 |
| > Inflection point | 0.99 (0.99, 1.00) 0.1660 |
| Log-likelihood ratio | <0.001 |

Age, race, education, marital status, PIR, BMI, smoking, drinking were adjusted.
